# Validation of the disease burden morbidity assessment by self-report in a French-speaking population

**DOI:** 10.1186/1472-6963-12-35

**Published:** 2012-02-14

**Authors:** Marie-Eve Poitras, Martin Fortin, Catherine Hudon, Jeannie Haggerty, José Almirall

**Affiliations:** 1 Département de médecine de famille, Université de Sherbrooke, Québec, Canada; 2 Centre de santé et de services sociaux de Chicoutimi, Québec, Canada; 3 Faculty of medicine, McGill University, Montreal, Canada; 4 Centre de santé et de services sociaux de Chicoutimi, 305 St-Vallier, Chicoutimi, Québec G7H 5H6, Canada

## Abstract

**Background:**

The Disease Burden Morbidity Assessment (DBMA) is a self-report questionnaire used to estimate the disease burden experienced by patients. The aim of this study was to test and to measure the properties of the French translation of the DBMA (DBMA-Fv).

**Methods:**

The original version of the DBMA was translated into French (Canadian) and first assessed during cognitive interviews. In the validation study, patients recruited during consecutive consultation periods completed the DBMA-Fv questionnaire while they were in the waiting room of a primary care setting (T1). Participants completed the same questionnaire mailed to their home two weeks later (T2). Concomitant validity of the DBMA-Fv was assessed using the Cumulative Illness Rating Scale (CIRS). Patient medical records were reviewed to verify chronic diseases and past medical history.

**Results:**

Ninety-seven patients were recruited and 85 (88%) returned the mailed questionnaires; 5 (5.9%) were incomplete. DBMA-Fv scores of the 80 participants with a complete questionnaire at T2 ranged from 0 to 30 (median 5.5, mean 7.7, SD = 7.0). Test-retest reliability of the DBMA-Fv was high (ICC: 0.86, 95% CI: 0.79-0.92). The DBMA-Fv and the CIRS correlated moderately at T1 (r = 0.46, 95% CI: 0.26 - 0.62, *p *< 0.01) and T2 (r = 0.56, 95% CI: 0.38 - 0.70, *p *< 0.01). The mean (SD) sensitivity of patient reports of a condition in relation to chart review at T2 was 73.9 (8.4) (range 62.5% to 90%). The overall mean (SD) specificity was 92.2 (6.7) (range 77.6% to 98.6%).

**Conclusions:**

The DBMA-Fv's properties are similar to its English counterpart as to its median sensitivity and specificity compared to chart reviews. It correlated moderately with an established index of multimorbidity. A high percentage of patients were able to complete the test correctly as a mail questionnaire and it showed high test-retest reliability.

## Background

Studies on multimorbidity should rely on valid and robust measurement to assess the disease burden experienced by patients with chronic diseases. Previous studies have shown that a measure that includes a weighting for severity is a better predictor of patient-related outcomes than a measure based on a simple disease count [1,2]. Severity can be judged on purely clinical grounds by health professionals or on the basis of the illness experienced by patients themselves. However, impact on daily living seems to be best evaluated by the patient because self-reported disease burden correlates with quality of life outcomes more strongly than measures of comorbidity based on other methods of data collection [3]. The Disease Burden Morbidity Assessment (DBMA) is a self-report questionnaire that seems promising in this respect [3]. There are many instruments designed to measure multimorbidity, such as the Cumulative Illness Rating Scale (CIRS) [4], the Charlson index [5], the Index of Coexistent Disease [6], and the Shwartz index [7]. However, these indexes have to be administered by professionals because of the medical background required to complete them. This is a limitation to using these instruments in large samples of patients either in primary care settings or the general population. The DBMA does not have this limitation. The DBMA is easily completed by older people and original validity assessment revealed good sensitivity and specificity using the chronic disease list in the medical chart as a gold standard [3,8]. Test-retest reliability and concomitant validity of the instrument have not been reported yet. The aim of this study was to test and to measure the properties of the French translation of the DBMA (DBMA-Fv). Specifically, we were interested in test-retest reliability, concomitant validity with another measure of multimorbidity and to explore the criterion diagnostic validity (sensitivity/specificity) with a gold standard.

## Methods

### The instrument

The DBMA is a self-report questionnaire in which the patient must rate a number of medical conditions, if present. The original version of the DBMA included 23 common medical conditions [3]. The instrument was later modified, and the number of conditions was reduced to 21 [8]. The instrument used for this study was the latter version with an additional item exploring depression/anxiety. In the original article of Bayliss and colleagues [3], depression was assessed as a separate outcome measure but due to its importance in patients with multimorbidity [9] we decided to include it as the 22^nd ^item. For each condition present, the patient assesses the degree to which the condition limits his/her daily activities on a five-point descriptive scale in which the first level, "not at all", has a weight of 1, and the fifth level, "a lot", has a weight of 5; all other conditions are scored zero. The questionnaire also allows patients to add medical conditions not included in the list and to score them in the same way. The total score is the sum of the limitation from all conditions, including those added by the patient. The questionnaire is written in a simple language understandable to patients using short sentences. The English version of the questionnaire is shown in more detail in an additional file (see Additional file 1).

### Translation

The original version of the DBMA [8] was translated in French Canadian following a procedure inspired by Vallerand [10] and Hébert [11]. Although forward-backward translation is a method commonly used for translating questionnaires in the field of patient-reported outcomes, given the simple nature of the questions we estimated that this procedure was not necessary. A bilingual translator translated the original version into French. A panel of experts examined both versions and made revisions to further adapt it to Quebec French. The translated version (DBMA-Fv) was then submitted to a panel of experts (three physicians and one nurse). The panel verified that the disease list of the instrument faithfully reflected the English version, and that the language was well adapted to the one used by patients seen in primary care. If any discrepancy was found, modifications and specifications were made to the questionnaire following expert recommendations.

### Cognitive interview

The DBMA-Fv was first assessed during cognitive interviews to make sure all items were clearly written, without ambiguity, and in a language that could easily be understood by the target population. To achieve this, two of the authors (MF and CH) recruited a convenience sample, as recommended by Dillman [12], from their consulting patients. The sample included 10 patients (7 women) aged from 19 to 79 years (mean ± SD, 63.3 ± 16.9) suffering from various chronic diseases, in a clinical practice. Patients agreeing to participate provided written consent. At each interview, an observer was also present. The interviewer and the observer were research assistants trained by one of the authors of the study (MEP). At the time of the interview, participants were asked to read the questions of the DBMA-Fv out loud and to express any thoughts or doubts they had, or to highlight ambiguities they may find while answering the questions. The interviewer led the activity while the observer had the task to note all the questions and comments of the participants. The maximum duration of interviews was 30 minutes.

After each interview, the interviewer and the observer met three authors of the study (MEP, MF and CH) in order to discuss the comments expressed by the participant. Questions identified as unclear were clarified by the research team for the next participant and so on until no further change was required. The questionnaire, modified according to participant input, was then considered to be in its final format and applied in the validation study. The final version of the DBMA-Fv validated in this study is shown in more detail in an additional file (see Additional file 2).

### Validation study

The patients were recruited by a research assistant during consecutive consultation periods from the waiting room of the Family Medicine Unit (FMU) of a regional health centre (Centre de santé et de services sociaux de Chicoutimi) in Saguenay (Quebec) Canada. We aimed to recruit a sample of 100 participants to test the instrument [12]. Patients solicited were asked to provide written consent and to complete a short questionnaire to determine eligibility. Participants had to be at least 18 years old, patients at the FMU for more than two years and able to read and write in French. Pregnant women, patients with an unstable acute condition, or having an uncontrolled psychiatric disease, a cognitive disorder, or unable to provide informed consent were not included in the study. Eligible patients were asked to complete the DBMA-Fv questionnaire without assistance while they were in the waiting room of the FMU (T1). Filling out the questionnaire took no more than 15 minutes. Participants agreed to complete the same questionnaire sent to their homes two weeks later (T2) and provided consent to the research team to access their medical records.

A second copy of the questionnaire along with a letter inviting participants to answer was sent to those who did not return the questionnaire one week after the date of the first mailing. Participants who did not answer the questionnaire one month after their recruitment were called up by the research coordinator of the study to point out the importance of their participation or to accept their withdrawal from the study.

A trained research nurse reviewed the medical records and completed a data extraction grid, including the list of chronic diseases and past medical history of each participant. This data was used to complete, first, a DBMA-Fv based on chart review and also to score the CIRS, another multimorbidity index [4], to assess concomitant validity. The measurement properties of the CIRS and the validity of this method for scoring the CIRS have been described elsewhere [1,13,14]. Briefly, the CIRS uses a scoring system that encompasses 14 anatomical domains (cardiac, vascular, hematological, respiratory, ophthalmologic-otorhinolaryngologic, upper gastrointestinal, lower gastrointestinal, hepatic-pancreatic, renal, genitourinary, musculoskeletal-tegument, neurological, endocrine-metabolic-breast, and psychiatric) and assigns a value from 0 (no impairment of the organ or system) to 4 (extremely severe impairment that is a life-threatening condition) to determine a severity score for each domain. In the case of multiple conditions affecting a particular domain, the condition with the highest score determines the score given to the domain. The global score is the sum of each domain's score.

### Data analysis

Missing data were analyzed by number of incomplete questionnaires and by unanswered items in the questionnaire. Questionnaires with one missing value or more were considered incomplete. To evaluate the test-retest reliability, we calculated the intraclass correlation coefficient (ICC) of the total score of the DBMA-Fv. After checking for normality of the distributions, the Pearson correlation was used to measure the concomitant validity of the DBMA-Fv compared to the CIRS. The 95% confidence interval (CI 95%) for the correlation coefficient of the DBMA-Fv vs. CIRS relationship at T1 and T2 was calculated using Fisher's Z transformation [15]. Analyses of sensitivity and specificity with a 95% CI were carried out for each medical condition included in the DBMA-Fv when numbers were sufficient. The 'gold standard' used to calculate sensitivity and specificity was the information contained in the medical records. These analyses compared the questionnaires completed by patients to those completed by the research nurse using patient charts. Since the DBMA was originally developed as a mail survey, we used the results of the mail questionnaire at T2 to assess diagnostic validity. Conditions found in an insufficient number of subjects (five or less) to provide a good estimation of the sensitivity were not included. Ill-defined diagnoses (back pain, stomach problem, colon problem, poor circulation) unlikely to be found in the chart review were also excluded from the estimation of sensitivity.

The SPSS 16.0 Software was used for data analysis. Questionnaires with missing values were excluded from analyses of test-retest reliability and concomitant validity. Approval for this project was obtained from the ethics committee of the Centre de santé et de services sociaux de Chicoutimi in February 2009.

## Results

A total of 100 patients were invited to participate in the validation study. Of those, 97 accepted and were eligible. The majority of participants were women (Table 1). At T1, nineteen patients (19.6%) did not answer some items and the questionnaire was considered incomplete. In total, 31 items were not answered and, among them, in 19 cases the medical condition was not reported in the chart either. Missing values did not cluster around any one disease. At T2, the total of unanswered items was only five (6%), i.e. one unanswered question in five patients, and in two cases, the medical conditions were not present in the chart. Significant differences between patients who completed the questionnaire correctly and those who did not were observed at T1 for age, CIRS score, education and marital status (Table 1). However, in a multivariate logistic regression model, age (*p *< 0.05) was the most important factor associated with an incomplete questionnaire; sex (*p *= 0.37), education (*p *= 0.1), and CIRS score (*p *= 0.78) were not important.

**Table 1:** Characteristics of the Sample at T1

Characteristic	Complete questionnaires (n = 78)	Incomplete questionnaires (n = 19)	*P* Value
Mean (SD) age in years	47.4 (15.9)	57.6 (13.6)	0.01*
Male%	32.1	36.8	0.32^†^
Mean (SD) CIRS score	6.6 (4.0)	8.8 (5.4)	0.04*
Education%			< 0.01^†^
< 8 y	5.1	10.5	
8 to 12 y	24.4	47.4	
> 12 y (college or university)	70.5	42.1	
Household income in Canadian			0.95^†^
dollars,%			
< $29,999	24.3	26.4	
≥ $30,000	71.9	73.6	
Marital status, %			0.02^†^
Married	73.1	63.2	
Divorced/Separated	9.0	15.8	
Widowed	3.8	10.5	
Single	14.1	10.5	

* *t*-test.

^†^Chi-square test.

Taking into account the DBMA-Fv responses at T2 (less unanswered items), the most frequent conditions reported by the patients were elevated cholesterol (32.9%), overweight (32.1%), stomach problem (30.6%), hypertension (26.2%), and osteoarthritis (25.9%). The conditions for which patients reported that the condition limited their daily activities "a lot" were other rheumatic diseases (14.3% of patients with the condition), back pain (10% of patients with the condition), angina/coronary artery disease (8.3% of patients with the condition), hard of hearing (7.1% of patients with the condition), and stomach problem (3.8% of patients with the condition). Four patients added medical conditions not included in the list: two patients added allergies, one patient added Raynaud's syndrome, and one patient added tinnitus. These conditions were not considered in the validation analyses.

### Reliability

At T1, DBMA-Fv scores of the 78 participants who correctly completed the questionnaire ranged from 0 to 27 (median 5.0, mean 6.4, SD = 6.1). At T2, of the 97 mailed questionnaires, 85 (88%) were returned by the patients. Among these, 5 (5.9%) were incomplete. DBMA-Fv scores of the 80 participants with a complete questionnaire at T2 ranged from 0 to 30 (median 5.5, mean 7.7, SD = 7.0). Test-retest reliability of the DBMA-Fv was high (ICC: 0.86, 95% CI: 0.79-0.92) among the 66 questionnaires with complete responses at T1 and T2.

### Validity

Concomitant validity: Scores of the CIRS ranged from 0 to 19 (median 7, mean 7.1, SD 4.3). The DBMA-Fv and the CIRS correlated moderately at T1 (r = 0.46, 95% CI: 0.26 - 0.62, *p *< 0.01) and T2 (r = 0.56, 95% CI: 0.38-0.70, *p *< 0.01). We also calculated the concomitant validity by considering only the questionnaires with complete data at T1 and T2 and obtained similar results (N = 66 (r = 0.45, 95% CI: 0.23 - 0.62, *p *< 0.01) and at T2 (r = 0.54, 95% CI: 0.34 - 0.69, *p *< 0.01).

Diagnostic validity: Table 2 reports the sensitivity and specificity measures at T2 calculated with the complete questionnaires. The gold standard used was the diagnosis of the condition obtained from the chart review. The mean ± SD sensitivity of patient reports of a condition in relation to chart review at T2 was 73.9 ± 8.4 (range 62.5% to 90%). The overall mean ± SD specificity was 92.2 ± 6.7 (range 77.6% to 98.6%).

**Table 2:** Sensitivity and specificity of the DBMA-Fv in relation to chart review at T2

Disease	TP + FN*	TP	Sensitivity %	CI 95%*	TN + FP*	TN	Specificity %	CI 95%	Prevalence % (n) †
Hypertension	26	20	77.0	56.4 - 91.0	57	55	96.5	87.9 - 99.6	31.3 (83)
Elevated cholesterol	32	26	81.2	63.6 - 92.8	52	50	96.2	86.8 - 99.5	38.1 (84)
Asthma	10	9	90.0	55.5 - 99.7	74	71	95.9	88.6 - 99.2	11.9 (84)
Diabetes	12	9	75.0	42.8 - 94.5	72	71	98.6	92.5 - 100	14.3 (84)
Osteoarthritis	21	14	66.7	43.0 - 85.4	63	55	87.3	76.5 - 94.4	25.0 (84)
Rheumatic disease, other	22	15	68.2	45.1 - 86.1	62	56	90.3	80.1 - 96.4	26.2 (84)
Overweight	16	12	75.0	47.6 - 92.7	67	52	77.6	65.8 - 86.9	19.3 (83)
Angina/coronary artery disease	16	10	62.5	35.4 - 84.8	67	65	97.0	89.6 - 99.6	19.3 (83)
Depression/Anxiety	13	9	69.2	38.6 - 90.9	71	64	90.1	80.7 - 95.9	15.5 (84)

* TP = True Positive; FN = False Negative; TN = True Negative; FP = False Positive; CI 95% = 95% Confidence Interval.

† Total n = 84 (one chart could not be retrieved). Differences between the total n and the actual n used in calculations in some diseases are due to incomplete data in the questionnaires that were not considered in the calculation.

## Discussion

The results of our study suggest that the DBMA-Fv provides a good estimate of the disease burden of patients seen in primary care. The properties of the DBMA-Fv are similar to its English counterpart as to its median sensitivity and specificity compared to chart reviews, and its correlation with a multimorbidity index which is sensitive to quality of life outcomes. In addition, it has good test-retest reliability. It accounts for many chronic diseases commonly seen in primary care practice. Previous studies have shown associations between DBMA score and quality of life, age, 'compound effects of conditions' (treatments and symptoms interfering with each other), self-efficacy (confidence in managing one's medical conditions), financial constraints, and physical functioning [3,8]. The DBMA-Fv provides an alternative for multimorbidity measurement in studies based on a self-report survey design.

Estimated for the first time, the test-retest reliability of the DBMA-Fv was satisfactory and similar to previous reports of comparable instruments [14,16].

The DBMA-Fv significantly correlated with the CIRS, a well-validated index of multimorbidity. The correlation was good but not perfect which is expected from instruments based on different constructs. The CIRS was designed to be completed during a clinical assessment, all diseases are evaluated, and scorers are required to have an appropriate background to complete the scale, whereas the DBMA is a self-report measure of disease burden.

Apart from the built-in evaluation of the impact on daily living, the DBMA-Fv is pretty simple to use and comparable to other questionnaires used in large population surveys [17], but shorter and easier to administrate. It could be used as a simple count of chronic conditions. Using the same list of conditions in different studies would allow better comparisons between them. The instrument can also be used as a count weighted for its impact on daily living for each condition.

The 'gold standard' used is considered a good reference point. However, with this study we could only explore the diagnostic validity of the DBMA as it did not have the statistical power to produce accurate estimates of sensitivity (true sick among those self-declared as sick) mainly because of the low prevalence of many conditions included in this questionnaire among the subjects of our sample. This resulted in large confidence intervals that precluded a reliable interpretation in many instances [18]. However, specificities (true negatives among those declaring not being sick) were more precise as there were higher numbers involved in the count. Nevertheless, in this study, mean specificity was higher (92.2%) than mean sensitivity (73.9%) which concurs with the report of the original version in which sensitivities and specificities were also calculated in relation to chart review [3].

Finding a sensitivity that is lower than the specificity suggests that patients under-reported conditions present in the medical chart [3]. This under-reporting may reflect a tendency to ignore diagnoses that are of less importance to them or even denied [3,19,20].

In this study, almost 20% of the questionnaires were incomplete at T1 (waiting room survey). Nonetheless, missing values did not cluster around any one disease, suggesting that overall the disease list was clearly understood. Distractions in the waiting room may have accounted for a higher rate of incomplete questionnaires at T1. Significant differences were found between patients who correctly completed the questionnaire and those who did not. Age was the most important factor in our analysis. As multimorbidity increases with age, it can be thought that the effect of age is due to a more demanding task when completing the DBMA in older patients. However, multimorbidity measured with the CIRS was not an important factor in the multivariate logistic regression model. Nevertheless, the number of incomplete questionnaires was reduced to 6% at T2 using the mail questionnaire. A mail questionnaire is thus more appropriate for this instrument.

This study has limitations. Statistical power is the most important. Generalizability may be limited by the characteristics of the population studied, a relatively small sample of patients consulting and composed predominantly of women. The small sample size in this study was sufficient for a good estimation of reliability and concomitant validity but resulted in a lack of precision for criterion validity and sensitivity in particular. In addition, we assumed that the presence of a diagnosis in patient charts was a 'gold standard' in the assessment of sensitivity and specificity, and this may not always be the case. Some medical conditions are more likely to be recorded in the chart, mainly those for which medications are prescribed. However, charts may be less accurate for recording conditions that patients are less likely to seek medical help for from their family doctor (hearing loss for example). For sensitivity, in most cases the confidence interval is very wide due to the small number of observations. Indeed, there were only three diseases (hypertension, elevated cholesterol and asthma) in which the 95% CI did not include the value of 50% and therefore, showed more precision. The test-retest reliability was analyzed using the 66 questionnaires with complete responses at T1 and T2 and this might be a select group within our sample. Also, the test-retest reliability was assessed using a mixed-mode approach (first test administered in the waiting room and the second test at home) which may have negatively influenced the results. Nevertheless, the ICC was high.

In conclusion, the French translation of the DBMA is a self-report estimate of disease burden that a high percentage of patients were able to complete correctly as a mail questionnaire. It can be used in studies involving primary care settings or the general population. It has high test-retest reliability and correlated moderately with an established index of multimorbidity, the CIRS. The DBMA-Fv showed an adequate diagnostic sensitivity, which needs to be further studied in a larger sample of subjects, and a very good diagnostic specificity. The instrument is a subjective multimorbidity measure that incorporates disease severity and explores the interference of medical conditions with patients' daily activities.

## Competing interests

The authors declare that they have no competing interests.

## Authors' contributions

MF and M-EP conceived and designed the study. They also supervised the data collection, analyzed the data and drafted the manuscript. JA, JH, and CH participated in the data analysis, and helped draft the manuscript. All authors read and gave their final approval of the version of the manuscript submitted for publication. M-EP takes responsibility for the integrity of the work as a whole.

## Pre-publication history

The pre-publication history for this paper can be accessed here:

http://www.biomedcentral.com/1472-6963/12/35/prepub

## Supplementary Material

Additional file 1 **English version of the DBMA**. Click here for file

Additional file 2 **French version of the DBMA**. Click here for file
