# Supplementary material for: Validation of the disease burden morbidity assessment by self-report in a French-speaking population
Source: BMC Health Serv Res. 2012 Feb 14;12:35. doi: 10.1186/1472-6963-12-35 (PMC3305524; doi:10.1186/1472-6963-12-35)
Supplement: Additional file 1 — English version of the DBMA. [file 1472-6963-12-35-S1.PDF]

Additional file 1
English version of the questionnaire

QUESTIONNAIRE ABOUT YOUR HEALTH CONDITION

INSTRUCTIONS

This questionnaire evaluates your health condition. It contains a list of 22 chronic health problems. Please, answer to the best of your knowledge. If you have a health problem; you must think about how the problem affects your daily activities. Note: you should be as specific as possible.

Do not forget to include treated diseases or those that are controlled for. It could be helpful to think of the medications that you currently take or treatments that you have received.

NB. The expression “daily activities” refers to the activities that a person of your age takes part in every day.

Thanks for your collaboration.

1. Do you suffer from hypertension (high blood pressure)? : ☐ NO ☐ YES

<u>If yes</u>, how much is this problem limiting you in your daily activities?
Limitation :
☐ Not at all ☐ A little ☐ Somewhat ☐ Quite a bit ☐ A lot

2. Do you suffer from a cholesterol problem? : ☐ NO ☐ YES

<u>If yes</u>, how much is this problem limiting you in your daily activities?
Limitation :
☐ Not at all ☐ A little ☐ Somewhat ☐ Quite a bit ☐ A lot

3. Do you suffer from asthma? ☐ NO ☐ YES

<u>If yes</u>, how much is this problem limiting you in your daily activities?
Limitation :
☐ Not at all ☐ A little ☐ Somewhat ☐ Quite a bit ☐ A lot

4. Do you suffer from a pulmonary problem (chronic bronchitis or emphysema)? : ☐ NO ☐ YES

<u>If yes</u>, how much is this problem limiting you in your daily activities?
Limitation :
☐ Not at all ☐ A little ☐ Somewhat ☐ Quite a bit ☐ A lot

5. Do you suffer from diabetes? : ☐ NO ☐ YES

<u>If yes</u>, how much is this problem limiting you in your daily activities?
Limitation :
☐ Not at all ☐ A little ☐ Somewhat ☐ Quite a bit ☐ A lot

6. Do you suffer from thyroid disorder? ☐ NO ☐ YES

<u>If yes</u>, how much is this problem limiting you in your daily activities?
Limitation :
☐ Not at all ☐ A little ☐ Somewhat ☐ Quite a bit ☐ A lo

Questions 7 to 11 refer to diseases that affect the spine (vertebral column) and joints. The disease has to have been diagnosed by a doctor to answer YES to a question.

<u>Note: Be careful not to answer YES to more than one question to indicate one disease</u>

7. Do you suffer from osteoarthritis? : ☐ NO ☐ YES

<u>If yes</u>, how much is this problem limiting you in your daily activities?
Limitation :
☐ Not at all ☐ A little ☐ Somewhat ☐ Quite a bit ☐ A lot

8. Do you suffer from rheumatoid arthritis? ☐ NO ☐ YES

<u>If yes</u>, how much is this problem limiting you in your daily activities?
Limitation :
☐ Not at all ☐ A little ☐ Somewhat ☐ Quite a bit ☐ A lot

9. Do you suffer from persistent back pain or sciatic pain (excluding osteoarthritis)? : ☐ NO ☐ YES

<u>If yes</u>, how much is this problem limiting you in your daily activities?
Limitation :
☐ Not at all ☐ A little ☐ Somewhat ☐ Quite a bit ☐ A lot

10. Do you suffer from osteoporosis? ☐ NO ☐ YES

<u>If yes</u>, how much is this problem limiting you in your daily activities?
Limitation :
☐ Not at all ☐ A little ☐ Somewhat ☐ Quite a bit ☐ A lot

11. Do you suffer from ANOTHER illness that affects the members or the articulations for more than 6 months (example: tendonitis, bursitis, fibromyalgia, lupus, etc)? ☐ NO ☐ YES

<u>If yes</u>, how much is this problem limiting you in your daily activities?
Limitation :
☐ Not at all ☐ A little ☐ Somewhat ☐ Quite a bit ☐ A lot

12. Do you suffer from reflux or heartburn or peptic ulcer? ☐ NO ☐ YES

<u>If yes</u>, how much is this problem limiting you in your daily activities?
Limitation :
☐ Not at all ☐ A little ☐ Somewhat ☐ Quite a bit ☐ A lot

13. Do you suffer from an intestine problem (example: irritable bowel syndrome, Crohn disease, ulcerative colitis, diverticulosis, etc)? ☐ NO ☐ YES

<u>If yes</u>, how much is this problem limiting you in your daily activities?
Limitation :
☐ Not at all ☐ A little ☐ Somewhat ☐ Quite a bit ☐ A lot

14. Do you suffer from a circulatory problem in your legs? ☐ NO ☐ YES

<u>If yes</u>, how much is this problem limiting you in your daily activities?
Limitation :
☐ Not at all ☐ A little ☐ Somewhat ☐ Quite a bit ☐ A lot

15. Do you suffer from overweight? ☐ NO ☐ YES

<u>If yes</u>, how much is this problem limiting you in your daily activities?
Limitation :
☐ Not at all ☐ A little ☐ Somewhat ☐ Quite a bit ☐ A lot

16. Do you suffer from an audition problem (hard of hearing)? ☐ NO ☐ YES

<u>If yes</u>, how much is this problem limiting you in your daily activities?
Limitation :
☐ Not at all ☐ A little ☐ Somewhat ☐ Quite a bit ☐ A lot

17. Do you suffer from a vision problem even though you wear glasses? ☐ NO ☐ YES

<u>If yes</u>, how much is this problem limiting you in your daily activities?
Limitation :
☐ Not at all ☐ A little ☐ Somewhat ☐ Quite a bit ☐ A lot

18. Do you suffer from cardiac illnesses (e.g.: angina, infarction, dilation, artery bypass, angioplasty, etc)? ☐ NO ☐ YES

<u>If yes</u>, how much is this problem limiting you in your daily activities?
Limitation :
☐ Not at all ☐ A little ☐ Somewhat ☐ Quite a bit ☐ A lot

19. Did you suffer from a CVA (cerebrovascular accident)? ☐ NO ☐ YES

<u>If yes</u>, how much is this problem limiting you in your daily activities?
Limitation :
☐ Not at all ☐ A little ☐ Somewhat ☐ Quite a bit ☐ A lot

20. Do you suffer from heart failure (diagnosis confirmed by your doctor)? ☐ NO ☐ YES

<u>If yes</u>, how much is this problem limiting you in your daily activities?
Limitation :
☐ Not at all ☐ A little ☐ Somewhat ☐ Quite a bit ☐ A lot

21. Did you suffer from a cancer in the 5 last years (including melanoma, but excluding all other skin cancers)? ☐ NO ☐ YES

<u>If yes</u>, how much is this problem limiting you in your daily activities?
Limitation :
☐ Not at all ☐ A little ☐ Somewhat ☐ Quite a bit ☐ A lot

22. Do you suffer from depression *or* anxiety problems?

☐ NO ☐ YES

<u>If yes</u>, how much is this problem limiting you in your daily activities?
Limitation :

☐ Not at all ☐ A little ☐ Somewhat ☐ Quite a bit ☐ A lot

Do you suffer from ANOTHER *or* MANY OTHER chronic health problems That weren't mentioned above? ☐ NO ☐ YES

<u>If yes</u>, name them and indicate how much each problem is limiting you in your daily activities:

1. ____________________

☐ Not at all ☐ A little ☐ Somewhat ☐ Quite a bit ☐ A lot

2. ____________________

☐ Not at all ☐ A little ☐ Somewhat ☐ Quite a bit ☐ A lot

3. ____________________

☐ Not at all ☐ A little ☐ Somewhat ☐ Quite a bit ☐ A lot

-End of the questionnaire. Thanks for your collaboration-
