# Supplementary material for: Validation of the disease burden morbidity assessment by self-report in a French-speaking population
Source: BMC Health Serv Res. 2012 Feb 14;12:35. doi: 10.1186/1472-6963-12-35 (PMC3305524; doi:10.1186/1472-6963-12-35)
Supplement: Additional file 2 — French version of the DBMA. [file 1472-6963-12-35-S2.PDF]

Additional file 2
French version of the questionnaire

QUESTIONNAIRE SUR VOTRE ÉTAT DE SANTÉ

INSTRUCTIONS

Ce questionnaire évalue votre état de santé. Il contient 22 problèmes de santé chroniques. Répondez au mieux de vos connaissances. Si vous avez ce problème de santé, vous devez réfléchir à comment ce problème affecte vos activités de la vie quotidienne. Attention, vous devez être le plus précis possible.

N'oubliez pas d'inclure les maladies traitées ou contrôlées. Pour vous aider, vous pouvez penser aux médicaments que vous prenez ou aux traitements que vous avez reçus.

N.B. L'expression « activités de la vie quotidienne » fait référence aux activités qu'une personne de votre âge fait tous les jours.

Merci de votre collaboration

1. Souffrez-vous d'hypertension (haute pression) ? : ☐ NON ☐ OUI

<u>Si OUI</u>, à quel point CE problème vous limite-t-il dans vos activités de la vie quotidienne ?
Limitation :
☐ Aucune ☐ Légère ☐ Moyenne ☐ Grande ☐ Très grande

2. Souffrez-vous d'un problème de cholestérol ? : ☐ NON ☐ OUI

<u>Si OUI</u>, à quel point CE problème vous limite-t-il dans vos activités de la vie quotidienne ?
Limitation :
☐ Aucune ☐ Légère ☐ Moyenne ☐ Grande ☐ Très grande

3. Souffrez-vous d'asthme ? ☐ NON ☐ OUI

<u>Si OUI</u>, à quel point CE problème vous limite-t-il dans vos activités de la vie quotidienne ?
Limitation :
☐ Aucune ☐ Légère ☐ Moyenne ☐ Grande ☐ Très grande

4. Souffrez-vous d'une maladie pulmonaire (bronchite chronique *ou* emphysème) ? ☐ NON ☐ OUI

<u>Si OUI</u>, à quel point CE problème vous limite-t-il dans vos activités de la vie quotidienne ?
Limitation :
☐ Aucune ☐ Légère ☐ Moyenne ☐ Grande ☐ Très grande

5. Souffrez-vous de diabète ? : ☐ NON ☐ OUI

<u>Si OUI</u>, à quel point CE problème vous limite-t-il dans vos activités de la vie quotidienne ?
Limitation :
☐ Aucune ☐ Légère ☐ Moyenne ☐ Grande ☐ Très grande

6. Souffrez-vous d'un trouble de la glande thyroïde ? ☐ NON ☐ OUI

<u>Si OUI</u>, à quel point CE problème vous limite-t-il dans vos activités de la vie quotidienne ?
Limitation :
☐ Aucune ☐ Légère ☐ Moyenne ☐ Grande ☐ Très grande

Les questions 7 à 11 se rapportent aux maladies qui touchent les articulations et la colonne vertébrale. Pour répondre oui à une question, il faut que la maladie ait été diagnostiquée par un médecin.

<u>Attention de ne pas répondre oui à plusieurs questions pour signifier une seule maladie</u>

7. Souffrez-vous d'arthrose ? : ☐ NON ☐ OUI

<u>Si OUI</u>, à quel point CE problème vous limite-t-il dans vos activités de la vie quotidienne ?
Limitation :
☐ Aucune ☐ Légère ☐ Moyenne ☐ Grande ☐ Très grande

8. Souffrez-vous de polyarthrite rhumatoïde ? ☐ NON ☐ OUI

<u>Si OUI</u>, à quel point CE problème vous limite-t-il dans vos activités de la vie quotidienne ?
Limitation :
☐ Aucune ☐ Légère ☐ Moyenne ☐ Grande ☐ Très grande

9. Souffrez-vous de douleurs au dos persistantes *ou* de douleurs sciatiques (excluant l'arthrose)? : ☐ NON ☐ OUI

<u>Si OUI</u>, à quel point CE problème vous limite-t-il dans vos activités de la vie quotidienne ?
Limitation :
☐ Aucune ☐ Légère ☐ Moyenne ☐ Grande ☐ Très grande

10. Souffrez-vous d'ostéoporose ? ☐ NON ☐ OUI

<u>Si OUI</u>, à quel point CE problème vous limite-t-il dans vos activités de la vie quotidienne ?
Limitation :
☐ Aucune ☐ Légère ☐ Moyenne ☐ Grande ☐ Très grande

11. Souffrez-vous d'une AUTRE maladie affectant les membres ou les articulations depuis plus de 6 mois (exemple : tendinite, bursite, fibromyalgie, lupus, etc.) ? ☐ NON ☐ OUI

<u>Si OUI</u>, à quel point CE problème vous limite-t-il dans vos activités de la vie quotidienne ?
Limitation :
☐ Aucune ☐ Légère ☐ Moyenne ☐ Grande ☐ Très grande

12. Souffrez-vous de reflux *ou* d'ulcères *ou* de brûlements d'estomac ? ☐ NON ☐ OUI

Si OUI, à quel point CE problème vous limite-t-il dans vos activités de la vie quotidienne ?
Limitation :
☐ Aucune ☐ Légère ☐ Moyenne ☐ Grande ☐ Très grande

13. Souffrez-vous d'une maladie de l'intestin (exemple : côlon irritable, maladie de Crohn, colite ulcéreuse, diverticulose, etc.) ? ☐ NON ☐ OUI

Si OUI, à quel point CE problème vous limite-t-il dans vos activités de la vie quotidienne ?
Limitation :
☐ Aucune ☐ Légère ☐ Moyenne ☐ Grande ☐ Très grande

14. Souffrez-vous d'un problème de circulation dans vos jambes ? ☐ NON ☐ OUI

Si OUI, à quel point CE problème vous limite-t-il dans vos activités de la vie quotidienne ?
Limitation :
☐ Aucune ☐ Légère ☐ Moyenne ☐ Grande ☐ Très grande

15. Souffrez-vous d'un surplus de poids ? ☐ NON ☐ OUI

Si OUI, à quel point CE problème vous limite-t-il dans vos activités de la vie quotidienne ?
Limitation :
☐ Aucune ☐ Légère ☐ Moyenne ☐ Grande ☐ Très grande

16. Souffrez-vous d'un problème d'audition (difficulté à entendre) ? ☐ NON ☐ OUI

Si OUI, à quel point CE problème vous limite-t-il dans vos activités de la vie quotidienne ?
Limitation :
☐ Aucune ☐ Légère ☐ Moyenne ☐ Grande ☐ Très grande

17. Souffrez-vous d'un problème de vision malgré le port de lunettes ? ☐ NON ☐ OUI

Si OUI, à quel point CE problème vous limite-t-il dans vos activités de la vie quotidienne ?
Limitation :
☐ Aucune ☐ Légère ☐ Moyenne ☐ Grande ☐ Très grande

18. Souffrez-vous d'une maladie cardiaque (ex. : angine, infarctus, dilatation, pontage, angioplastie, etc.) ? ☐ NON ☐ OUI

Si OUI, à quel point CE problème vous limite-t-il dans vos activités de la vie quotidienne ?
Limitation :
☐ Aucune ☐ Légère ☐ Moyenne ☐ Grande ☐ Très grande

19. Avez-vous souffert d'un AVC (accident vasculaire cérébral) ? ☐ NON ☐ OUI

Si OUI, à quel point CE problème vous limite-t-il dans vos activités de la vie quotidienne ?
Limitation :
☐ Aucune ☐ Légère ☐ Moyenne ☐ Grande ☐ Très grande

20. Souffrez-vous d'insuffisance cardiaque (diagnostic confirmé par votre médecin) ? ☐ NON ☐ OUI

Si OUI, à quel point CE problème vous limite-t-il dans vos activités de la vie quotidienne ?
Limitation :
☐ Aucune ☐ Légère ☐ Moyenne ☐ Grande ☐ Très grande

21. Avez-vous souffert d'un cancer <u>dans les 5 dernières années</u> (incluant les mélanomes, mais excluant tous les autres cancers de la peau) ? ☐ NON ☐ OUI

Si OUI, à quel point CE problème vous limite-t-il dans vos activités de la vie quotidienne ?
Limitation :
☐ Aucune ☐ Légère ☐ Moyenne ☐ Grande ☐ Très grande

22. Souffrez-vous d'une dépression *ou* d'un problème d'anxiété ?

☐ NON ☐ OUI

Si OUI, à quel point CE problème vous limite-t-il dans vos activités de la vie quotidienne ?
Limitation :

☐ Aucune ☐ Légère ☐ Moyenne ☐ Grande ☐ Très grande

Souffrez-vous d'un AUTRE ou de PLUSIEURS AUTRES problèmes de santé chroniques qui n'ont pas été mentionnés plus haut ? ☐ NON ☐ OUI

Si OUI, nommez-les et dites à quel point chaque problème vous limite dans vos activités de la vie quotidienne :

1. ___________________

☐ Aucune ☐ Légère ☐ Moyenne ☐ Grande ☐ Très grande

2. ___________________

☐ Aucune ☐ Légère ☐ Moyenne ☐ Grande ☐ Très grande
1#

3. ___________________

☐ Aucune ☐ Légère ☐ Moyenne ☐ Grande ☐ Très grande

-Fin du questionnaire. Merci de votre collaboration-
